# Citrus flavonoids mitigate the cisplatin-induced ovarian toxicity via dual modulation of Nrf2/HO-1 pathway and NF-κB axis

**DOI:** 10.3389/fphar.2026.1903801

**Published:** 2026-07-17

**Authors:** Ahmed Abdeen, Obeid Shanab, Mohammed H. Hassan, Hassan Ahmed, Dania Abdelhady, Ibrahim A. Mostafa, Eman A. Elgebaly, Heba A. Elnoury, Mona K. Alawam, Mustafa Shukry, Samah F. Ibrahim, Sherif A. Shaltout, Nermin Afify, Ekramy M. Elmorsy

**Affiliations:** 1 Department of Forensic Medicine and Toxicology, Faculty of Veterinary Medicine, Benha University, Toukh, Egypt; 2 Department of Biochemistry, Faculty of Veterinary Medicine, Qena University, Qena, Egypt; 3 Department of Medical Biochemistry and Molecular Biology, Faculty of Medicine, Qena University, Qena, Egypt; 4 Department of Physiology, Faculty of Veterinary Medicine, Qena University, Qena, Egypt; 5 Department of Biomedical Sciences, College of Medicine, Dubai Medical University, Dubai, United Arab Emirates; 6 Department of Physiology, Faculty of Medicine, Benha University, Benha, Egypt; 7 Department of Forensic Medicine and Clinical Toxicology, Faculty of Medicine, Benha University, Benha, Egypt; 8 Department of Pharmacology, Faculty of Medicine, Benha University, Benha, Egypt; 9 Department of Biomedical Sciences, College of Veterinary Medicine, King Faisal University, Saudi Arabia, Al-Ahsa, Saudi Arabia; 10 Department of Internal Medicine, College of Medicine, Princess Nourah bint Abdulrahman University, Riyadh, Saudi Arabia; 11 Department of Clinical Pathology, Student Hospital Shebin Al-Kom, Menoufia University, Shebin AlKom, Egypt; 12 Department of Community Health, Faculty of Nursing, Al-Baha University, Al-Baha, Saudi Arabia; 13 Center for Health Research, Northern Border University, Arar, Saudi Arabia

**Keywords:** cell survival, cytoprotection, inflammatory cytokines, nutraceuticals, redox homeostasis, steroidogenesis

## Abstract

Cisplatin (CIS) is an effective chemotherapeutic agent whose clinical use is limited by off-target toxicity, including damage to ovarian granulosa cells, characterized by oxidative stress and inflammation. Here, the cytoprotective potential of the citrus flavonoids, naringin (NG) and hesperidin (HP), was investigated in ovarian granulosa cells exposed to CIS-induced toxicity. Molecular docking demonstrated that NG and HP exhibited strong binding affinities for key antioxidant, nuclear factor erythroid 2–related factor 2 (Nrf2) and catalase (CAT) as well as anti-apoptotic B-cell lymphoma-2 (Bcl-2) proteins, while CIS preferentially interacted with pro-apoptotic targets. *In vitro*, CIS dose-dependently reduced cell viability and hormone secretion (progesterone and estradiol), and these reductions were significantly restored by co-treatment with NG or HP. CIS-induced oxidative injury, marked by elevated accumulation of reactive oxygen species (ROS), lipid peroxidation, and depletion of reduced glutathione (GSH), as well as key antioxidant defenses (superoxide dismutase (SOD) and catalase (CAT), was effectively mitigated by the NG and HP. Furthermore, NG and HP suppressed the CIS-triggered inflammation by inhibiting NF-κB activation and downregulating the release of major pro-inflammatory mediators (tumor necrosis factor-alpha (TNF-α), interleukin (IL)-6, IL-8, IL-1β). They also counteracted CIS-induced apoptosis by reducing phosphorylation of protein kinase B (Akt), cytochrome c (Cyt c) release, caspase (Cas) activation, and the Bcl-2-associated X protein (*Bax*)/*Bcl-2* ratio. These findings demonstrated that NG and HP could protect ovarian granulosa cells from CIS-induced damage through dual regulation of the Nrf2/heme oxygenase-1 (*HO-1*) antioxidant and nuclear factor kappa B (*NF-κB*) inflammatory axis, highlighting their potential as adjuvant therapies to mitigate chemotherapy-induced ovarian toxicity.

## 1 Introduction

Cisplatin (CIS) has been used for decades as a front-line chemotherapeutic drug for treating a variety of tumors because of its potent ability to reduce carcinogenesis and metastasis (Ranasinghe et al., 2022). Despite its efficacy, the biological applications of CIS are constrained by significant toxicity in various organs, including nephrotoxicity (Pabla and Dong, 2008), neurotoxicity (Sałat, 2020), ototoxicity (Li et al., 2023a), and myelosuppression (Romani, 2022). Additionally, CIS adversely affects the ovaries, particularly in women of reproductive age (Li et al., 2023b). The toxicity of CIS is mostly due to its ability to cause oxidative stress in normal cells, rendering it hazardous (Ibrahim et al., 2021). This results in the reduction of antioxidant enzyme activity, as well as an overabundance of ROS, leading to lipid peroxidation, protein misfolding, and DNA adduct formation, as well as disrupting mitochondrial function, eventually leading to necroptosis (Elgazzar et al., 2022; Elsafty et al., 2022; Habotta et al., 2023; Li et al., 2023b; Mapuskar et al., 2023). Furthermore, CIS-induced oxidative stress triggers signaling pathways to produce pro-inflammatory cytokines, thus worsening tissue injury and CIS toxicity (Ramkumar et al., 2021; Mentese et al., 2022). Therefore, antioxidant supplementation may be an effective treatment to halt tissue damage caused by CIS.

Naringin (NG) and hesperidin (HP), bioactive flavonoids present in citrus fruits, have attracted considerable attention for their multiple pharmacological actions, particularly their abilities to counteract oxidative stress, inflammation, and apoptosis (Hassan et al., 2021). Previous reports suggested that these compounds exert strong antioxidant activity by directly neutralizing ROS and stimulating endogenous antioxidant systems. These compounds have been reported to exhibit strong antioxidant activity through direct neutralization of ROS and stimulation of endogenous antioxidant systems. The protective effect of this compound is mainly due to activation of the Nrf2 signaling pathway and increased transcription of cytoprotective enzymes, including HO-1. The anti-inflammatory activity is primarily due to the inhibition of the NF-κB cascade, which in turn downregulates pro-inflammatory mediators, such as TNF-α and interleukins (Rodríguez-García et al., 2019; Stabrauskiene et al., 2022). The dual effects on oxidative damage and the suppression of inflammatory responses indicate their pharmacological potential for the treatment of oxidative stress-related chronic inflammatory diseases. A large number of studies have highlighted the effectiveness of NG in ameliorating oxidative damage and inflammation associated with various diseases and different toxicants, including diclofenac toxicity (Hassan et al., 2021) and Alzheimer’s disease (Kuşi et al., 2024) and the effectiveness of HP in indomethacin toxicity (Jain and Parmar, 2011) and pleurisy (Adefegha et al., 2017).

Our critical review of the existing literature revealed an important gap, as to the best of our knowledge, no previous studies have directly examined the cytoprotective potential of NG or HP against CIS-induced ovarian damage. Therefore, we investigated the potential protective effects of NG and HP in an *in vitro* model of ovarian granulosa cells against CIS-induced damage and apoptosis. Pro-inflammatory responses, cell viability, apoptosis, and oxidative stress biomarkers were measured. Molecular docking was used to study the binding affinities of CIS, NG, and HP to key oxidative stress, inflammatory, and apoptotic proteins, to better understand the toxicity of CIS and the protective effects of NG and HP.

## 2 Materials and methods

### 2.1 Molecular docking

In this study, the molecular docking was carried out to analyze the interactions of CIS, NG, and HP with the key proteins in oxidative stress, inflammatory signaling and apoptotic pathways, such as Nrf2 (PDB ID: 5CGJ), HO-1 (PDB ID: 1N3U), IL-6 (PDB ID: 4ni9), IL-8 (PDB ID: 1IL8), NF-κB (PDB ID: 4DN5), Bax (PDB ID: 4S0O), Bcl-2 (PDB ID: 6O0K), Cas-3 (PDB ID: 2XYG), CAT (PDB ID: 1QQW), and SOD (PDB ID: 1SPD). The 3D structures of the target proteins were obtained from the Protein Data Bank (PDB). The chemical structures of CIS, NG, and HP were retrieved from the PubChem database and energy minimized before docking. The protein structures were prepared by removing water molecules and co-crystallized ligands, and by adding polar hydrogen atoms. Protein structures were prepared by removing water molecules and co-crystallized ligands, and adding polar hydrogen atoms. Molecular docking simulations were carried out using the SwissDock web server based on the EADock DSS algorithm, and binding affinities were calculated using the AutoDock Vina scoring function. Docking was performed using standard parameters and the binding pose with the lowest predicted free binding energy (ΔG, kcal/mol) was selected for further analysis. SwissDock’s integrated visualization tools were used to visualize interaction patterns and binding affinity (Bugnon et al., 2024). The method revealed the binding processes of the ligand-protein complexes, active-site interactions and therapeutic potential.

### 2.2 Cell culture preparation

At 37 °C, the KGN human ovarian granulosa cell line (CVCL_0375, Cellcook Biotech, China) was cultivated in DMEM/F-12 media, 1% penicillin/streptomycin (Beyotime, China), 10% (vol: vol) charcoal-stripped fetal bovine serum (VivaCell), 5% CO_2_, and lacking phenol red. Upon receipt, cells were expanded and routinely passaged at 70%–80% confluence; only cells from passages 3-10 were used for all experiments. The cell line was obtained from a commercial supplier with authentication records, and cultures were routinely monitored for *mycoplasma* contamination using standard detection procedures to maintain mycoplasma-free cell cultures throughout the study. Following cell adhesion, fresh culture medium was replaced every other day to ensure optimal growth conditions.

### 2.3 MTT assay

KGN cells were cultivated until they achieved 80%–90% confluence. After that, for 48 h, cells were treated with either NG or HP (10, 20, 40, 80, 160 µM), or CIS (1, 5, 10, 15, 20 µM). The assay used a commercial kit and was written according to the manufacturer’s instructions (Sigma-Aldrich, USA). Finally, absorbance was measured at 590 nm using a TopCount microplate reader (Perkin Elmer, Ueberlingen, Germany). The experiment was performed once more with extra cotreatments. Later, cells were incubated with CIS (5 or 10 µM) in the presence or absence of NG or HP (80 µM) for 48 h. Each experiment was performed in three independent replicates.

### 2.4 Determination of inflammatory cytokines using ELISA assays

Cytokine concentrations, specifically TNF-α (Catalog No. ab208348), IL-6 (Catalog No. ab208113), IL-8 (Catalog No. ab214297), IL-1β (Catalog No. ab108768), and NF-κB p65 protein (Catalog No. ab191440), were measured using ELISA kits (Abcam, UK), based on the manufacturer’s protocol. Briefly, standards and samples were added to pre-coated 96-well plates and incubated under the manufacturer’s specified conditions. The plates were then sequentially incubated with the detection antibody and enzyme-linked conjugate, with thorough washing steps between each stage to minimize non-specific binding. Absorbance was quantified at 450 nm utilizing a microplate reader (BioTek Instruments, USA). A standard curve was generated from absorbance values of known cytokine concentrations to quantify cytokine levels in the samples. Cytokine concentrations were calculated from the standard curve using a four-parameter logistic (4-PL) regression model. All samples were analyzed in technical triplicate and biological triplicate to ensure reproducibility and reliability of the results.

### 2.5 Evaluation of oxidative status

For oxidative stress investigations, cells were cultured and treated with CIS (5 or 10 µM) for 48 h, with or without NG or HP (80 µM). Next, all measurements regarding the ROS, lipid peroxidation, GSH, and antioxidant enzymes were performed.

#### 2.5.1 Measuring the ROS production

The ROS assay used a dichlorofluorescein diacetate (DCFDA) solution (25 µM in Hank’s solution), as described by Elmorsy et al. (2014). The fluorescent probe 2′,7′-dichlorofluorescein diacetate (DCFDA; Sigma-Aldrich) enabled the quantification of intracellular ROS levels in KGN granulosa cells. A flow cytometer, such as the BD FACSCanto II, uses excitation/emission wavelengths of 488/530 nm to quantify fluorescence intensity. The FlowJo software (Tree Star Inc., USA) was employed for data administration. An increase in dichlorofluorescein fluorescence may signify elevated intracellular ROS levels. In parallel, fluorescence signals were monitored at excitation and emission wavelengths of 485 and 535 nm, respectively. At each time point, all measurements were conducted in triplicate.

#### 2.5.2 Assessment of lipid peroxidation

Thiobarbituric acid reactive substances (TBARS), especially malondialdehyde (MDA), were determined by a commercial assay kit (Catalog Number: ab118970, Abcam) according to the manufacturer’s instructions. In short, samples were reacted with thiobarbituric acid under high-temperature acid conditions to form a chromogenic MDA-TBA adduct and extracted for spectrophotometric quantification. Absorbance was measured at 532 nm. Each experiment was carried out in biological triplicate with technical replicates to ensure reproducibility and analytical reliability.

#### 2.5.3 Evaluation of cellular antioxidants

The activity of CAT (Catalog No. ab83464) and SOD (Catalog No. ab65354) was measured using Abcam assay kits according to the manufacturer’s instructions. In short, enzymatic activities were measured according to the kit instructions by monitoring absorbance/fluorescence changes spectrophotometrically arising from the specific catalytic conversion of the substrates supplied in each kit. The GSH levels were determined using the ELISA Kit (Fluorometric-Green, ab138881, Abcam) following the manufacturer’s instructions. The content of reduced glutathione (GSH) was determined by a fluorometric reaction with a thiol-specific probe, yielding a stable fluorescent signal proportional to GSH concentration. To prevent GSH oxidation, every precaution was taken to minimize exposure to light and air. All assays were performed in biological triplicate and technical replicate to ensure analytical reproducibility and data reliability.

### 2.6 Apoptosis assays

Cas-3, Cas-8, and Cas-9 were quantified using fluorometric assay kits (Takara Bio Inc., Clontech Laboratories, USA) according to the manufacturer’s instructions (Catalog Nos. 631,231, 631,225, and 631,228, respectively). Cas activity was assayed by cleavage of specific fluorogenic peptide substrates that release a fluorescent reporter proportional to enzyme activity using a microplate fluorometer at the recommended excitation/emission settings. All assays were done in biological triplicate and technical replicates per condition to ensure accuracy and reproducibility.

### 2.7 Assessment of Cyt c release into the cytosol

Following the manufacturer’s instructions, cytosolic Cyt c concentrations were measured using the Cyt c Release Assay Kit (Cat. No. ab65311, Abcam, UK). The kit’s ice-cold Cytosol Extraction Buffer was used to homogenize the tissue samples, which were then incubated on ice. Centrifugation of the homogenates at 700 × g for 10 min at 4 °C was used to sediment nuclei and cellular debris. To separate the cytosolic fraction and precipitate mitochondria, the supernatants were centrifuged at 10,000 × g for 30 min at 4 °C. The obtained cytosolic fractions were used to quantify Cyt c. Following the manufacturer’s instructions, a particular ELISA test was used to determine the amount of cytosolic Cyt c. A microplate reader was used to measure absorbance at 450 nm. The corresponding standard curve was used to determine the concentrations of Cyt c. The activation of the intrinsic apoptotic pathway and permeabilization of the mitochondrial outer membrane were thought to be indicated by an increase in cytosolic Cyt c.

### 2.8 Evaluation of the Akt kinase activity

A commercially available assay kit (Abcam) was used to measure Akt kinase activity in accordance with the manufacturer’s instructions. After treatment, the culture medium was aspirated, and any remaining medium components were quickly removed from the cells by washing them with ice-cold phosphate-buffered saline. After that, the cells were scraped in the supplied lysis solution, which contained phosphatase and protease inhibitors to maintain kinase activity, and the lysates were collected. After lysis, the lysates were collected and centrifuged for 15 min at 13,000 rpm before analysis. To guarantee equal loading, the protein concentration of the cleared lysates was ascertained prior to the experiment. A microplate reader was used to measure the absorbance at 450 nm after the stop solution was introduced at the conclusion of the test. To ensure accurate results, the assay was performed in biological triplicate with technical replicates for each experimental condition.

### 2.9 Annexin V-FITC/PI apoptosis assay using flow cytometry

The Annexin V-FITC/PI death detection kit (Cat. No. V13242, Thermo Fisher Scientific, USA) was used to measure apoptotic cell death in accordance with the manufacturer’s instructions. KGN human ovarian granulosa cells were added to six-well plates, and the corresponding treatments were then applied. Following incubation, adherent and floating cells were collected, washed 3 times with cold PBS, and resuspended in binding buffer. Each sample was then incubated for 15 min at room temperature in the dark after 5 μL of Annexin V-FITC and 5 μL of propidium iodide were added. Samples were immediately examined using a flow cytometer (BD FACSCanto II from BD Biosciences, USA) after 400 µL of binding buffer was added. The FlowJo application (Tree Star Inc., Ashland, OR, USA) was used to collect and evaluate the data; cells were classified as necrotic (Annexin V–/PI+), early death (Annexin V+/PI–), late death (Annexin V+/PI+), or alive (Annexin V–/PI–).

### 2.10 Quantitative PCR

Following harvesting of the treated cells, total RNA was extracted using the RNeasy Mini Kit (Qiagen, Germany) according to the manufacturer’s instructions. Complementary DNA synthesis and quantitative real-time PCR (qRT-PCR) analyses were performed using a CFX96 Real-Time PCR Detection System (Bio-Rad Laboratories, USA). The primer sequences used in this study are listed in Supplementary Table S1. Glyceraldehyde-3-phosphate dehydrogenase (*GAPDH*) was used as the internal housekeeping gene to normalize gene expression. Relative mRNA expression levels were calculated using the 2^−ΔΔCT^ method as described by Livak and Schmittgen, (2001). All experiments were performed in triplicate.

### 2.11 Statistical analyses

Data analysis was performed using GraphPad Prism version 8.4 (GraphPad Software Inc., USA). First, all data were assessed for normality and homogeneity, and then group comparisons involving three or more conditions were assessed using one-way analysis of variance (ANOVA) with Tukey’s *post hoc* test for multiple comparisons. A p-value of less than 0.05 was considered indicative of statistical significance. In addition, multivariate analyses, including principal component analysis (PCA), clustering heatmap, correlation heatmap, and volcano plot, were generated using RStudio (version 2025.05.1).

## 3 Results

### 3.1 Molecular docking

**FIGURE 1:**
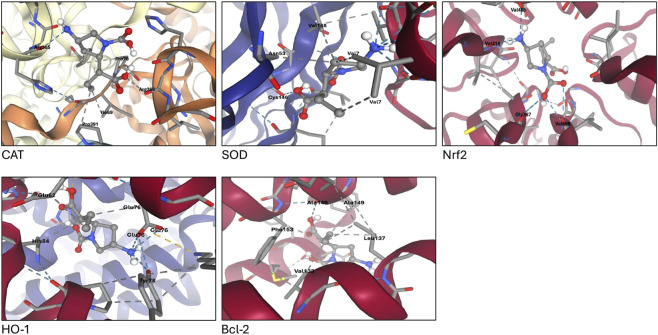
Molecular docking of cisplatin (CIS) with oxidative stress, inflammatory, and apoptotic key proteins. Binding affinities (kcal/mol) and interaction patterns are shown. Hydrogen bonds are depicted as dashed lines; hydrophobic contacts are presented as arcs. Computational docking was performed using SwissDock, and binding affinities were assessed through the AutoDock Vina scoring function.

The docking results for CIS revealed variable binding affinities and interaction features with five target proteins: CAT, SOD, Nrf2, HO-1, and Bcl-2 (Table 1; Figure 1). Nrf2 showed the highest binding affinity (−6.61 kcal/mol) with seven hydrogen bonds, mainly interacting with Gly367, Val418, Val465, and Val606. The SOD with binding affinity of −5.9 kcal/mol interacted with Val7, Asn53, and Cys146, forming four hydrogen bonds and six hydrophobic interactions.

**TABLE 1:** Molecular docking of cisplatin (CIS) with oxidative stress, inflammation, and apoptotic key proteins.

Target	Affinity (kcal/mol)	H-bonds	Hydrophobic contacts	Key interacting residues
CAT	−5.16	2	3	Pro391, Ile69, Arg365, Pro70
SOD	−5.90	4	6	Val7, Asn53, Cys146, Val148, Cys6
Nrf2	−6.61	7	0	Gly367, Val418, Val465, Val606
HO-1	−4.81	3	3	Tyr78, Glu66, His84, Glu62
Bcl-2	−5.00	2	5	Leu137, Val133, Phe153, Ala139, Asp111

CAT and Bcl-2 exhibited comparable hydrogen bonding and hydrophobic interactions, resulting in modest affinities of −5.16 and −5.0 kcal/mol, respectively. HO-1 had the lowest binding affinity (−4.81 kcal/mol) while maintaining significant interactions with Tyr78, Glu66, and His84. Overall, Nrf2 emerged as the most favorable binding target for CIS based on the lowest binding energy and extensive hydrogen-bonding network, suggesting its potential sensitivity to CIS-induced modulation. In contrast, HO-1 showed comparatively weaker interaction stability, indicating a lower likelihood of strong CIS binding.

**FIGURE 2:**
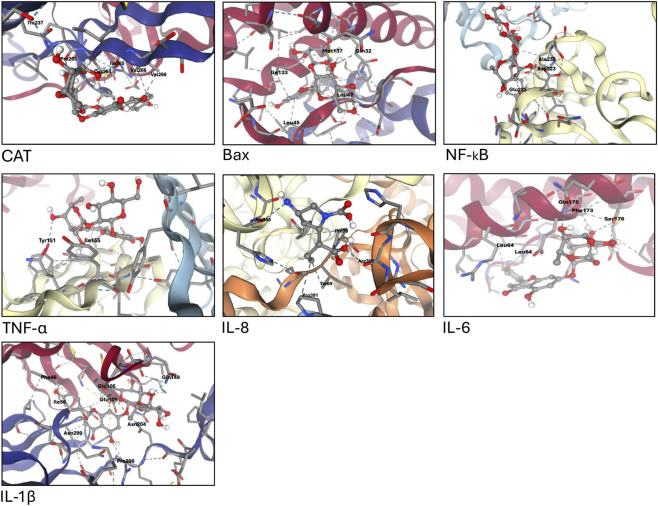
Molecular docking of naringin (NG) with oxidative stress, inflammatory, and apoptotic key proteins. Binding affinities (kcal/mol) and interaction patterns are shown. Hydrogen bonds are depicted as dashed lines; hydrophobic contacts are presented as arcs. Computational docking was performed using SwissDock, and binding affinities were assessed through the AutoDock Vina scoring function.

**FIGURE 3:**
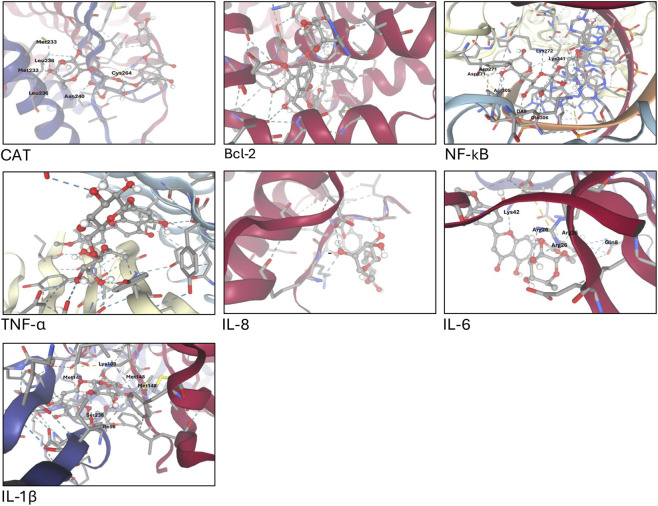
Molecular docking of hesperidin (HP) with oxidative stress, inflammatory, and apoptotic key proteins. Binding affinities (kcal/mol) and interaction patterns are shown. Hydrogen bonds are depicted as dashed lines; hydrophobic contacts are presented as arcs. Computational docking was performed using SwissDock, and binding affinities were assessed through the AutoDock Vina scoring function.

The docking data for the NG and HP ligands against various inflammatory and apoptotic targets are presented in Table 2 and Figures 2, 3 to elucidate binding efficiency and interaction patterns. With the help of the enhancement of hydrogen bonds and hydrophobic interactions, TNF-α had the highest binding affinity with both ligands. HP had a better binding (−8.5 kcal/mol) than NG (−7.67 kcal/mol). HP formed fewer hydrogen bonds than NG but showed higher binding affinity (−5.8 kcal/mol) for Cas-3 than NG (−5.37 kcal/mol). The interactions with key residues, such as Tyr197 and Met233, support a reliable binding mechanism. In general, HP showed higher binding affinity to important inflammatory and apoptotic targets, especially TNF-α and Cas-3, than NG, with a more stable interaction profile. Variations in binding patterns may contribute to the relative potency of HP in modulating inflammatory and apoptotic signaling pathways.

**TABLE 2:** Molecular docking of naringin (NG) and hesperidin (HP) with oxidative stress, inflammation, and apoptotic-key proteins.

Target	Ligand	Affinity (kcal/mol)	H-bonds	Hydrophobic contacts	Key interacting residues
Cas-3	NG	−5.37	8	3	Val266, Ile265, Cys265, Pro263, Thr237
	HP	−5.8	6	2	Met233, Leu236, Cys264, Asn240, Pro263, Tyr197, Val266
Bax	NG	23.5	7	6	Gln28, Leu27, Gln32, Leu45, Leu47, Ile133, Met137, Arg134
	HP	22.6	5	8	Gln28, Gly29, Gln32, Ala42, Ala46, Val50, Ile133, Leu141, Arg134, Gly138
NF-κB	NG	−4.26	2	3	Glu222, Asp223, Ala235
	HP	−6.8	5	3	DA5, Arg305, Asp271, Lys272, Lys241, Gln306
IL-8	NG	−5.84	4	2	Lys23, Glu24, Arg26, Ile28, Ser30
	HP	−4.95	8	0	Gln8, Lys42, Arg26
IL-6	NG	−4.5	5	1	Leu64, Phe173, Gln175, Ser176
	HP	−4.9	3	0	Leu64, Lys86, Glu93
IL-1β	NG	−5.64	3	6	Phe46, Ile56, Glu105, Gln149, Asn204, Pro206, Asn299
	HP	−2.9	6	3	Lys103, Met148, Ser238, Ile58
TNF-α	NG	−7.67	1	1	Tyr151, Ile155
	HP	−8.5	8	3	Ser60, Gln61, Tyr151, Tyr59, Tyr119, Leu120, Ile155, Leu157

In contrast, Bax exhibited positive binding energy values for both NG (+23.5 kcal/mol) and HP (+22.6 kcal/mol), indicating thermodynamically unfavorable interactions and poor ligand–protein compatibility. These findings suggest that Bax is unlikely to be a primary molecular target of NG and HP, and the results should therefore be interpreted with caution; the activation of apoptosis should be considered an outcome of oxidative stress, inflammation, and the interaction of apoptosis pathways.

### 3.2 Cytotoxicity of CIS, NG, or HP

**FIGURE 4:**
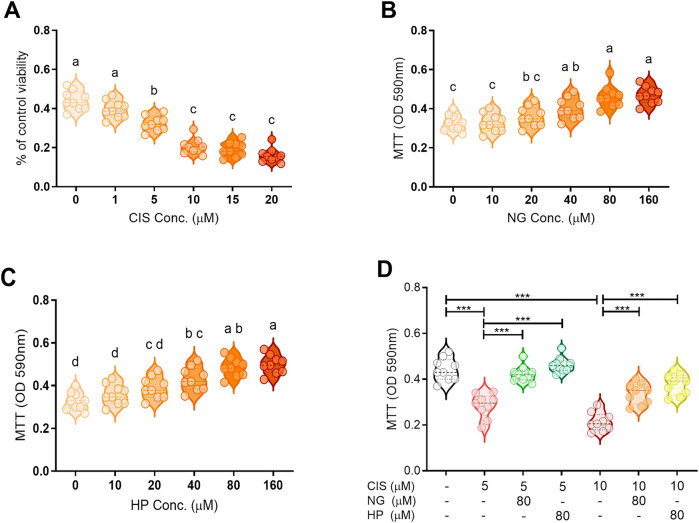
Violin-dot plots present the effect of cisplatin (CIS), naringin (NG), and hesperidin (HP) on granulosa cell viability and hormone secretion. **(A)** Dose-dependent effect of CIS (1–20 μM) on KGN cell viability measured by MTT assay. **(B)** Effect of NG (10–160 μM) on cell viability. **(C)** Effect of HP (10–160 μM) on cell viability. **(D)** Protective effect of NG (80 μM) or HP (80 μM) co-treatment against CIS-induced cytotoxicity at 5 and 10 μM. Data are presented as violin-dot plots with the mean (thick dotted line) ± SE (thin dotted line) from three independent experiments (n = 9). Statistical significance was evaluated using one-way analysis of variance (ANOVA) followed by Tukey’s post hoc test for multiple comparisons (***P < 0.001). Different lowercased-letters indicate significance between different concentration treatments at *P* < 0.05. Conc, concentration; CIS5, cisplatin (5 μM); CIS10, cisplatin (10 μM); NG, naringin (80 μM); HP, hesperidin (80 μM).

CIS has a dose-dependent cytotoxic effect, with a considerable reduction in viability at increasing concentrations (p < 0.05), as confirmed by the MTT assay (Figure 4A). NG increased (*p* < 0.05) cell viability at 40–160 µM doses, with a dose-dependent rise in MTT absorbance (Figure 4B). HP induced similar protective effects (Figure 4C). High HP concentrations significantly increase MTT absorbance (p < 0.05), indicating enhanced cell viability. The effect of co-treatment of CIS-exposed cells with NG or HP was displayed in Figure 4D and partially (*p* < 0.001) restored ovarian granulosa cells’ viability, indicating a protective role of these flavonoids.

### 3.3 Effects of CIS, NG, and HP on progesterone and estradiol levels

**FIGURE 5:**
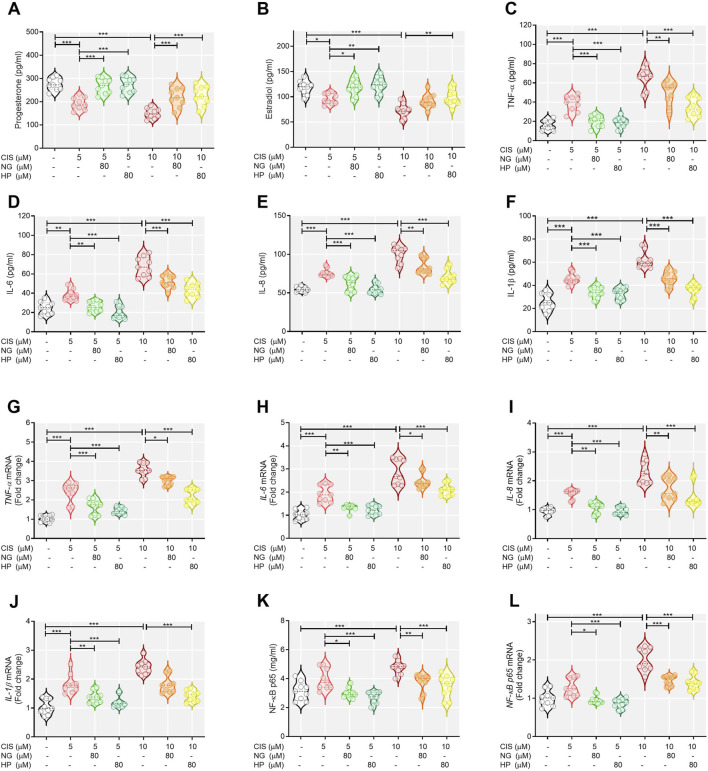
Progesterone **(A)** and estradiol **(B)** levels; activities of TNF-α **(C)**, IL-6 **(D)**, IL-8 **(E)**, and IL-1β **(F)**; mRNA expression levels of TNF-α **(G)**, IL-6 **(H)**, IL-8 **(I)**, and IL-1β **(J)**; and NF-κB p65 protein **(K)** and mRNA **(L)** levels in the granulosa cell homogenate. Data are presented as violin-dot plots with the mean (thick dotted line) ± SE (thin dotted line) from three independent experiments (n = 9). Statistical significance was evaluated using one-way analysis of variance (ANOVA) followed by Tukey’s post hoc test for multiple comparisons. *P < 0.05, **P < 0.01, ***P < 0.001. CIS5, Q15 cisplatin (5 μM); CIS10, cisplatin (10 μM); NG, naringin (80 μM); HP, hesperidin (80 μM).

As depicted in Figures 5A,B, Exposure to CIS at doses of 5 and 10 μM markedly reduced progesterone by 1.44-fold (*p* < 0.0001) and 1.84-fold (*p* < 0.0001), respectively, compared with the control group. In the CIS5 groups, NG80 and HP80 increased the parameter by 1.40-fold (*p* = 0.0001) and 1.42-fold (*p* < 0.0001), respectively, relative to the CIS5 group, restoring values to levels comparable with the control group (*p* > 0.99 for both comparisons). Similarly, in animals exposed to CIS10, administration of NG80 and HP80 significantly elevated the parameter by 1.47-fold (*p* = 0.0006) and 1.50-fold (*p* = 0.0002), respectively, compared with the CIS10 group.

Analysis of estrogen levels revealed a significant reduction following CIS exposure. Compared with the control group, estrogen levels decreased by 1.23-fold in the CIS5 group (*p* = 0.0315) and by 1.63-fold in the CIS10 group (*p* < 0.0001). Moreover, estrogen levels were significantly lower in the CIS10 group than in the CIS5 group (1.33-fold decrease, *p* = 0.0148), indicating a dose-dependent detrimental effect of CIS. Administration of NG80 and HP80 effectively restored estrogen levels in CIS-exposed animals. In the CIS5 groups, NG80 and HP80 increased estrogen levels by 1.23-fold (*p* = 0.0258) and 1.29-fold (*p* = 0.0029), respectively, compared with the CIS5 group. Notably, estrogen levels in both CIS5+NG80 and CIS5+HP80 groups were comparable to those of the control group (*p* > 0.9999 and *p* = 0.9826, respectively). Similarly, treatment with NG80 and HP80 increased estrogen levels in CIS10-exposed animals by 1.27-fold (*p* = 0.0768) and 1.37-fold (*p* = 0.0039), respectively, relative to the CIS10 group. No significant differences were observed between NG80 and HP80 treatments within either the CIS5 (*p* = 0.9894) or CIS10 (*p* = 0.9348) groups.

### 3.4 Effects of CIS, NG, and HP on the inflammatory response

Figures 5C–L shows that CIS (5 and 10 µM) dramatically induced secretion levels of the proinflammatory mediators TNF-α, IL-6, IL-8, and IL-1β, along with upregulation (*p* < 0.001) of their mRNA expression compared with control cells. These events were accompanied by activation of the NF-κB pathway, as indicated by marked increases (*p* < 0.001) in NF-κB p65 protein abundance and mRNA transcript levels relative to untreated cells. However, when CIS-exposed cells were co-treated with NG or HP, the CIS-induced inflammatory reaction was mitigated, as evidenced by significant (*p* < 0.001) reductions in proinflammatory cytokine levels and NF-κB p65 expression. These data suggested the anti-inflammatory potential of NG and HP.

### 3.5 Alterations in the oxidant/antioxidants balance in response to CIS, NG, and HP

**FIGURE 6:**
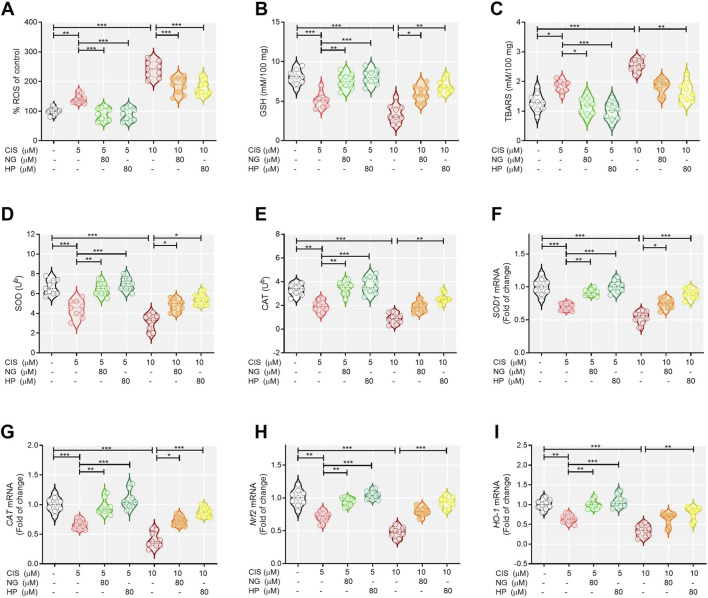
Violin-dot plots present the effect of cisplatin (CIS), naringin (NG), and hesperidin (HP) on the oxidative stress biomarkers in human granulosa cells. Levels of ROS **(A)**, GSH **(B)**, and TBARS **(C)**, and activities of SOD **(D)** and CAT **(E)**. mRNA expression levels of SOD1 **(F)**, CAT **(G)**, Nrf2 **(H)**, and HO-1 **(I)** in the granulosa cell homogenate. Data are presented as violin-dot plots with the mean (thick dotted line) ± SE (thin dotted line) from three independent experiments (n = 9). Statistical significance was evaluated using one-way analysis of variance (ANOVA) followed by Tukey’s *post hoc* test for multiple comparisons. *P < 0.05, **P < 0.01, ***P < 0.001. CIS5, cisplatin (5 µM); CIS10, cisplatin (10 µM); NG, naringin (80 µM); HP, hesperidin (80 µM).

As depicted in Figure 6, the current findings demonstrate the influence of CIS treatment on oxidative stress modulation by evaluating key antioxidant and oxidative damage indicators. CIS at low and high doses evoked an oxidative stress cascade indicated by dramatic increases (*p* < 0.05; *p* < 0.001) in the TBARS (lipid peroxidation marker) levels concurrently with depletion of the cellular antioxidant system involving decreases in GSH concentrations and CAT, SOD1, Nrf2, and *HO-1* mRNA expression levels in comparison to the control group (*p* < 0.01). These data might contribute to the notable declines in CAT and SOD enzymatic activity (*p* < 0.01). However, co-treatment with NG or HP significantly (*p* < 0.05) decreased MDA and increased GSH concentrations, and restored the reduced mRNA expression of *CAT, SOD1, Nrf2,* and *HO-1*, accompanied by increased (*p* < 0.05) CAT and SOD activities, compared with CIS-treated granulosa cells. These results demonstrated a robust antioxidant property of NG and HP.

### 3.6 Changes in the cell survival/death after treatment with CIS, NG, and HP

**FIGURE 7:**
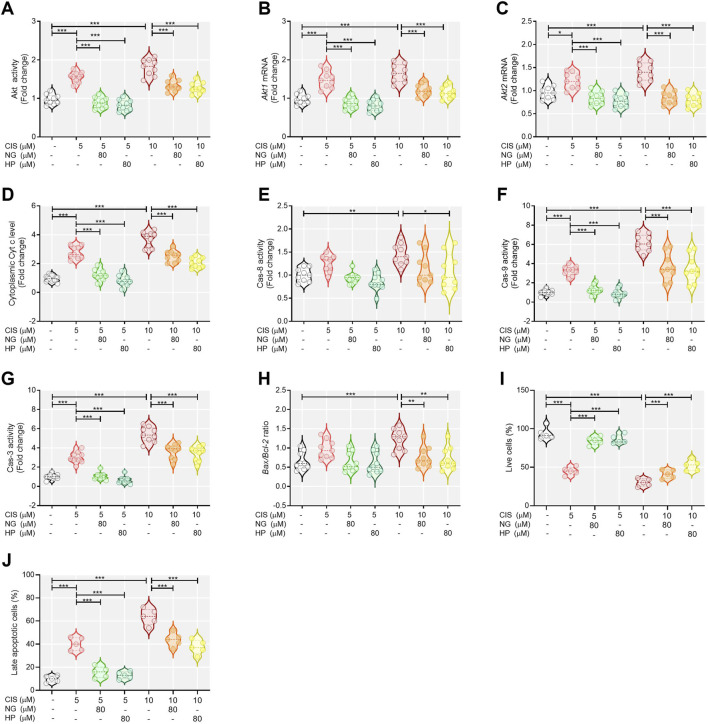
Violin-dot plots present the effect of cisplatin (CIS), naringin (NG), and hesperidin (HP) on cell survival and apoptotic markers in the human granulosa cells. Akt activity **(A)**, Akt1 mRNA **(B)**, Akt2 mRNA **(C)**, cytoplasmic Cyt c level **(D)**, Cas-8 activity **(E)**, Cas-9 activity **(F)**, Cas-3 activity **(G)** in the granulosa cell homogenate **(H)** *Bax/Bcl-2* ratio. Percentage of live cells **(I)**. Percentage of late apoptotic cells **(J)**. Data are presented as violin-dot plots with the mean (thick dotted line) ± SE (thin dotted line) from three independent experiments (n = 9). Statistical significance was evaluated using one-way analysis of variance (ANOVA) followed by Tukey’s *post hoc* test for multiple comparisons. *P < 0.05, **P < 0.01, ***P < 0.001. CIS5, cisplatin (5 µM); CIS10, cisplatin (10 µM); NG, naringin (80 µM); HP, hesperidin (80 µM).

The effects of CIS, NG, and HP on Akt activity and its coding genes, Akt1 and Akt2, were evaluated, as indicated in Figure 7. *Akt*-associated genes (*Akt1* and *Akt2*) were significantly upregulated (p < 0.001) by CIS alone compared with the control group. Consequently, CIS was shown to significantly (p < 0.001) increase Akt phosphorylation to 1.56 ± 1.2- and 1.84 ± 1.8-fold changes relative to control levels at concentrations of 5 and 10 µM, respectively, demonstrating Akt activation. On the other hand, Akt phosphorylation was markedly restored (p < 0.001) by co-treatment with NG and HP, suggesting a protective role in survival signaling.

Moreover, to evaluate the impact of CIS, NG, and HP on apoptotic and survival pathways in ovarian granulosa cells, cytoplasmic Cyt c, along with Cas-3, -8, and -9 levels and the *Bax/Bcl-2* ratio, was assessed. CIS treatments (5 and 10 µM) significantly (*p* < 0.001) increased Cyt c release into the cytoplasm compared with control levels (Figure 7). Furthermore, CIS (10 µM) considerably (*p* < 0.001) elevated the Bax/Bcl-2 ratio to 1.84 ± 3.3 (fold change) of the control level, supporting pro-apoptotic signaling. In parallel, the CIS-treated group had substantially higher (*p* < 0.01) activities of Cas-3, -8, and -9 in a dose-dependent manner. Conversely, co-therapy with NG or HP could significantly (*p* < 0.05) control cell death by inhibiting caspases, accompanied by downregulation of Cas activities and the *Bax/Bcl-2* ratio, suggesting modulation of apoptosis (Figure 7).

### 3.7 Multivariate analyses of CIS-induced cytotoxicity and therapeutic interventions

**FIGURE 8:**
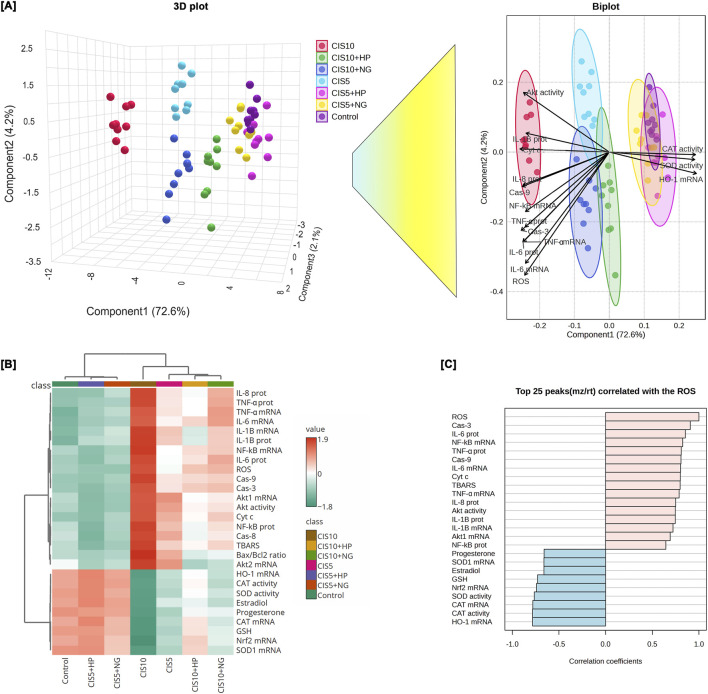
Multivariable data analyses. **(A)** Principal component analysis, PCA (left side; 3D plot, right side; biplot). **(B)** Clustering heatmap of the concentration averages. **(C)** Correlation heatmap exhibiting the top 25 variables ranked by their Pearson correlation coefficients with cellular ROS production. Red indicates positive correlations (variables that increase with ROS), while blue indicates negative correlations (variables that decrease as ROS increases). Plots were generated using RStudio (version 2025.05.1). CIS5, cisplatin (5 µM); CIS10, cisplatin (10 µM); HP, hesperidin; NG, naringin.

As indicated in Figure 8A, the 3D PCA revealed that the first three principal components collectively captured the majority of the dataset variability, accounting for 78.9% of the total variance. Notably, PC1 alone contributed the largest proportion of the variance (72.6%), enabling clear discrimination between the experimental groups. In contrast, the remaining components, PC2 and PC3, contributed minimally, explaining 4.2% and 2.1% of the variance, respectively. Furthermore, the PCA biplot (Figure 8A, right panel) revealed that markers of inflammation and oxidative injury were the main contributing variables that significantly separate the CIS-injured cells (clustered on the left side) from the control group. Furthermore, groups treated with CIS alone (CIS5, CIS10) clustered separately from those receiving combination therapies (CIS5+HP, CIS5+NG, CIS10+HP, CIS10+NG), suggesting that adjunct interventions with NG or HP markedly modulate CIS-induced cytotoxicity.

Additionally, the clustering heatmap (Figure 8B) included both treatment groups and the average concentration of the measured parameters. It exhibited distinct co-segregation patterns associated with unique treatment-specific molecular signatures. A clustering heatmap showed significant differences in the mean concentrations of the variables studied among the Control, CIS, CIS + NG, and CIS + HP groups. On the contrary, the Control, CIS + NG, and CIS + HP groups showed normal levels. The CIS group showed significant toxicity as indicated by significant changes in inflammatory and oxidative stress markers, apoptotic markers, cell survival markers, and hormonal levels. The experimental groups dendrogram showed a main bifurcation with a clear separation between the Control group and the CIS-treated cells. There was a substructure dose-dependent within the CIS cluster. The CIS10 group was its own branch, and the CIS5, CIS5+HP, and CIS5+NG groups were all pooled. The CIS10+HP and CIS10+NG groups created their own node.

The correlation heatmap presented in Figure 8C showed the top 25 variables significantly correlated with the cellular ROS production. These data indicated that proinflammatory cytokines and apoptotic markers were the most strongly positively correlated with ROS levels. In contrast, antioxidant markers and hormonal parameters exhibited the strongest negative correlations with ROS. Collectively, these findings highlight a clear inverse relationship between a pro-oxidant, inflammation- and apoptosis-associated molecular profile and a protective antioxidant-hormonal signature in relation to ROS accumulation.

**FIGURE 9:**
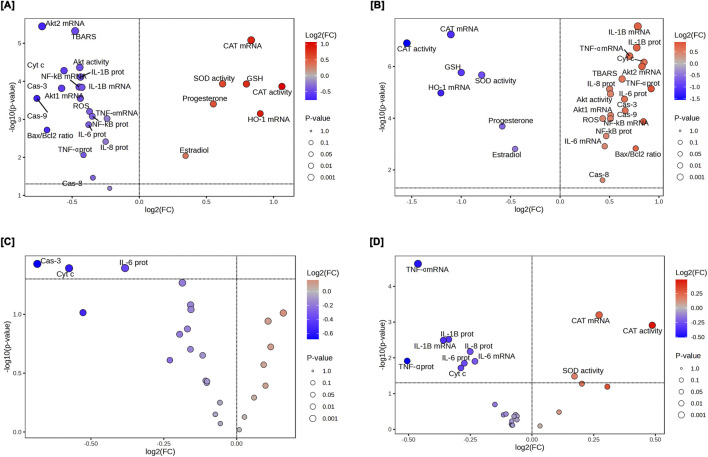
Volcano plots. **(A)** CIS10+NG vs*.* CIS10. **(B)** CIS10+HP vs*.* CIS10. **(C)** CIS5+HP vs*.* CIS5+NG. **(D)** CIS10+HP vs*.* CIS10+NG. Plots were generated using RStudio (version 2025.05.1). Significance was defined as log2 (fold change; FC) > 1 and -log10 (*p*-value) > 1.3 (equivalent to *p* < 0.05). CIS5, cisplatin (5 µM); CIS10, cisplatin (10 µM); HP, hesperidin; NG, naringin.

The volcano plot analysis revealed significant disparities in the measured parameters among different treatment groups (Figure 9). In the comparison between CIS10+NG and CIS10 (Figure 9A), NG co-treatment markedly enhanced antioxidant defense systems, including *CAT* mRNA expression, CAT enzymatic activity, *HO-1* mRNA levels, SOD activity, and GSH content, and increased progesterone and estradiol levels. At the same time, apoptotic and inflammatory mediators like Cyt c, caspases, ROS, TBARS, NF-κB, TNF-α, IL-1β, IL-6, and IL-8 were suppressed, suggesting the preventive action of NG against CIS-induced cytotoxicity at a higher dose of 10 µM. In the same line, CIS10+HP compared to CIS10 (Figure 9B) exhibited a greater protective profile, with higher antioxidant activity (CAT, SOD, HO-1, GSH) and a clear decrease in pro-inflammatory, cell survival, and apoptotic markers (Cyt c*,* caspases*, Akt*, NF-κB, TNF-α, ILs, TBARS). Figure 9C indicated that CIS5+HP exhibited only small alterations compared with CIS5+NG. These changes included small reductions in Cas-3 activity, Cyt c release, and IL-6 protein levels. On the other hand, a considerable number of significant parameters between CIS10+HP and CIS10+NG (Figure 9D) denoted by the enhancement of the antioxidant capacity, including increases in the CAT mRNA, CAT activity, and SOD activity, while also lowering the levels of several pro-inflammatory mediators (TNF-α, IL-1β, IL-6, IL-8, Cyt c). Collectively, these data attested to the therapeutic potential of HP, with HP outweighing NG supplementation, especially in the CIS10 model, as indicated by significant increases in antioxidant defenses and reductions in oxidative stress, apoptosis, and inflammatory signaling.

## 4 Discussion

Although CIS is effective against many cancers, it causes considerable ovarian damage. The mechanisms of this toxicity are inflammation, oxidative injury, and apoptosis (Mentese et al., 2022). Antioxidants, including melatonin and resveratrol, may reduce oxidative stress, inflammatory responses, and apoptosis in CIS-induced ovarian injury (Ibrahim et al., 2021; Al-Shahat et al., 2022). The current study investigated the protective effect of two citrus flavonoids, NG and HP. The concentrations used in this study for CIS (5 and 10 µM), NG (80 µM), and HP (80 µM) were consistent with previous studies, ensuring relevance and comparability with the literature. The cytotoxic and apoptotic effects of CIS have been well documented *in vitro* at low micromolar concentrations (1–10 µM), which represent the clinically achievable plasma concentrations in patients undergoing chemotherapy (Ibrahim et al., 2021; Li et al., 2023a). Similarly, 80 µM NG and HP have been previously used to investigate oxidative stress and cellular responses as this concentration causes significant biochemical changes but not immediate cell death (Alam et al., 2022; Danis et al., 2024). These concentrations enable a robust comparison of the differential effects of CIS, NG, and HP on oxidative damage and apoptotic pathways, providing a better understanding of their mechanistic roles. Granulosa cells are important for ovarian function, including folliculogenesis, steroidogenesis, and oocyte maturation, and thus are an important target for environmental and chemotherapeutic toxins (Sudhakaran et al., 2023; Hegazy et al., 2025). They are susceptible to oxidative stress and apoptosis, making them useful for studying mechanisms of ovarian toxicity and the protective effects of therapeutic interventions.

The present study demonstrated that CIS exerted cytotoxic effects on the human ovarian granulosa cell line, with inflammation, oxidative damage, and apoptosis identified as key underlying mechanisms. NG and HP, notable flavonoids derived from citrus fruits, are characterized by distinctive chemical structures that underpin their bioactive properties. NG possesses a flavanone backbone with a rhamnoside sugar moiety, while HP contains a rutinose group linked to the flavonoid core. These structural configurations are crucial for their bioactivity, enabling interactions with cellular enzymes and receptors involved in the regulation of inflammation, oxidative injury, and apoptotic pathways (Rehman et al., 2021). The hydroxyl groups in their molecular structures are essential for scavenging reactive substances and regulating cellular redox states (Li et al., 2021). NG and HP are positioned as essential substances for therapeutic investigation in the treatment of chronic disorders (Alam et al., 2022).

Basically, the molecular docking data not only predict binding events but also offer a structural framework that coherently explains the hierarchy of our biochemical results. The preferential binding of CIS to the Nrf2 provided a direct structural basis for the suppression of the antioxidant response, whereas the engagement of both flavonoids with the TNF-α and the NF-κB explains their robust anti-inflammatory actions at the molecular level. Importantly, the superior binding of HP over NG to TNF-α, NF-κB, and Cas-3 correlates well with the marginal but consistent superiority of HP in rescuing antioxidant enzyme activities and lowering inflammatory markers at the 10 µM CIS dose. Conversely, the lack of significant binding to Bax supports the notion that the anti-apoptotic effect of NG and HP is not mediated by direct inhibition of Bax but rather by upstream suppression of ROS-driven apoptotic signals and restoration of the Akt-mediated survival axis. These structural-functional correlations substantiate the proposed dual pathway model and reinforce the therapeutic relevance of citrus flavonoids as multitarget protective agents against chemotherapy-induced ovarian toxicity.

Consequently, the present results indicate that NG and HP exert potent anti-inflammatory effects by inhibiting pro-inflammatory cytokine production and attenuating NF-κB activation, thereby suppressing the CIS-induced inflammatory response. Suggest their potential therapeutic value in inhibiting chemo-induced inflammatory response. Both NG and HP have been shown to possess anti-inflammatory activities in experimental models *in vitro* and *in vivo*. The anti-inflammatory effects of NG and HP have been highlighted by growing evidence from previous studies, primarily attributed to the regulation of key inflammatory signaling pathways. These flavonoids reduce the production of pro-inflammatory mediators, including TNF-α, IL-1β, and IL-6, by inhibiting activation of the NF-κB and mitogen-activated protein kinase (MAPK) pathways, thereby mitigating inflammatory responses (Abharian et al., 2025; Ghaith et al., 2025; Alfawaz et al., 2026). HP has also been reported to inhibit NF-κB signaling, thereby reducing the production of NO, PGE2, and certain inflammatory cytokines. Such effects are associated with downregulation of iNOS and COX-2 expression, highlighting HP’s ability to interfere with critical molecular mediators involved in the propagation of inflammatory responses (Khan et al., 2026). HP protects human osteoarthritis chondrocytes against IL-1β-induced inflammation by reducing MMP-3 and MMP-13 production and suppressing NF-κB activation, thus preserving cartilage integrity (Fu et al., 2018).

NG, another citrus flavonoid, is anti-inflammatory and analgesic. Research shows that naringenin reduces pro-inflammatory mediators, including TNF-α, IL-1β, and IL-6, primarily by inhibiting NF-κB activation (Gad et al., 2025). In addition, NG activates the Nrf2 signaling pathway, leading to enhanced HO-1 expression and reduced oxidative stress (Alemdar et al., 2026). Further investigations have shown that naringenin can reduce inflammation by modifying immunological responses and suppressing ROS generation, thereby alleviating oxidative stress and limiting tissue injury (Solanki et al., 2025). Collectively, these findings highlight the therapeutic potential of HP and naringenin as natural anti-inflammatory agents, promising candidates for managing inflammatory diseases. Oxidative stress assays demonstrate that CIS significantly induces oxidative stress, leading to heightened lipid peroxidation, increased ROS generation, oxidative damage, and depletion of the antioxidant defense system. NG and HP effectively counteracted these alterations by enhancing the activities of endogenous antioxidant enzymes, restoring GSH levels, and reducing indices of oxidative damage. The results indicate that NG and HP exhibit significant antioxidant properties, potentially helping to reduce chemotherapy-induced oxidative injury. This agrees with previous data showing that both flavonoids possess significant antioxidative characteristics. In various experimental models of type 2 diabetes, HP and NG have been reported to improve antioxidant status by elevating glutathione content and antioxidant enzyme activities, while reducing oxidative stress biomarkers such as MDA and NO. These beneficial effects, together with their ability to suppress inflammatory cytokine production, suggest that both flavonoids exert protective actions against the onset and progression of diabetes-associated complications (Alghamdi, 2026; Pribadi et al., 2026).

HP and NG supplementation reduce oxidative stress in numerous clinical states, according to further research. In rats with diclofenac-induced hepatotoxicity, both flavonoids, alone and together, dramatically reduced serum levels of liver enzymes and proinflammatory cytokines and increased liver antioxidant enzyme activity. Histological analyses confirmed biochemical findings of reduced hepatic damage. NG and HP worked synergistically as antioxidants and anti-inflammatories (Hassan et al., 2021). NG and HP protect against hypertension, thrombosis, and other cardiovascular complications via their antioxidant and anti-inflammatory effects. (Adams et al., 2025). HP and NG enhance antioxidant activity by modulating lipid metabolism and inflammatory gene expression. Flavonoid supplementation enhanced meat fatty acid profiles and increased antioxidant defense gene expression in broilers, suggesting a role in reducing oxidative stress and inflammation (Hager-Theodorides et al., 2021). These studies demonstrate the antioxidant properties of HP and NG, suggesting their use in treating oxidative stress.

Cell survival, proliferation, and death depend on the PI3K/Akt signaling system. Ovarian granulosa cells require activation of this pathway for optimal follicular growth and function (Zheng et al., 2022). We found that CIS therapy increases Akt phosphorylation in healthy human ovarian granulosa cells, suggesting the unexpected activation of a survival pathway in response to CIS-induced apoptosis (Tang et al., 2001). However, studies indicate that chemotherapeutic drugs may cause PI3K/Akt pathway overactivation, leading to ovarian toxicity and follicular depletion (Ji et al., 2025). In contrast, NG and HP decreased Akt phosphorylation in these cells. Inhibition of Akt phosphorylation by NG affects cell survival and proliferation across diverse cell types (Cui et al., 2026). HP was also shown to inhibit Akt phosphorylation in various experimental models (Zhang et al., 2025). Our investigation found that NG and HP reduced Akt phosphorylation, suggesting that these flavonoids may inhibit activation of the CIS-induced survival pathway in granulosa cells. This effect of flavonoids may be more beneficial in cancer cells, facilitating CIS-induced apoptosis and increasing its therapeutic efficacy.

In the current study, NG and HP were found to significantly counteract CIS-induced apoptosis to variable extents. Prior studies showed that NG and HP demonstrated antiapoptotic actions by the modulation of mitochondrial-dependent pathways, suppression of oxidative stress and inflammatory responses, and modulation of key signaling networks involved in cell survival (Hassan et al., 2021; Ghaith et al., 2025). Such mechanisms support the potential therapeutic value of NG and HP in dysregulated apoptosis-related disorders by modifying apoptotic pathways.

It is worth noting that both flavonoids have been reported to promote apoptotic cell death in several cancer cell models (Contreras-Angulo et al., 2025; Tajaldini et al., 2025). This contradiction in their effects in action among healthy, immune, and cancer cells is mainly due to differences in molecular contexts and signaling pathways across these cell types. These flavonoids target dysregulated pathways in cancer cells, thereby promoting apoptotic cell death. For instance, NG has been reported to promote apoptotic cell death in cervical cancer cells by inhibiting the NF-κB/COX-2/Cas-1 signaling pathway. Many malignancies overactivate this pathway, boosting cell survival and proliferation. NG kills cancer cells by inhibiting this mechanism (Alhalmi et al., 2025). Healthy cells regulate apoptosis and do not signal abnormally as cancer cells do. Thus, NG and HP are less likely to promote normal cell death, reducing cytotoxicity. This selective effect shows that flavonoids can treat cancer cells without harming healthy tissue. Thus, NG and HP appear to selectively promote apoptosis in cancerous cells by regulating aberrant signaling pathways specific to malignant cells, indicating their potential as targeted anticancer treatments.

It is interesting to note that the mechanisms examined in this study inflammation, oxidative damage, and apoptosis are interrelated processes that impact cellular homeostasis and the genesis of illness. Oxidative stress, which leads to cellular and molecular damage, is encouraged by an imbalance between ROS production and antioxidant defenses. Furthermore, increased ROS levels can trigger NF-κB-dependent inflammatory signaling, which increases the synthesis of pro-inflammatory cytokines such interleukins and TNF-α (Ranneh et al., 2017). Additionally, ROS controls stress-sensitive MAPK pathways, including JNK. When JNK is activated, transcription factors like c-Jun are phosphorylated, which influences the production of apoptotic genes. JNK-mediated phosphorylation triggers the intrinsic apoptotic pathway by releasing pro-apoptotic proteins from mitochondria (Kannan and Jain, 2000). An essential component of this relationship is the endoplasmic reticulum (ER). Misfolded proteins build up and the unfolded protein response is triggered by ER stress, which is frequently caused by oxidative damage. The transcription factor C/EBP homologous protein (CHOP), which promotes apoptosis, can be expressed more when UPR activation is prolonged. Increased ROS production, oxidative stress, and apoptotic signaling result from CHOP’s upregulation of ER oxidoreduction 1 alpha (ERO1α) expression (Li et al., 2010; Ekundayo et al., 2024). Oxidative stress can also activate the NLRP3 inflammasome, a multiprotein complex essential to the innate immune response. Pro-inflammatory cytokines like IL-1β are generated when the NLRP3 inflammasome is active, connecting oxidative stress to inflammation (Li et al., 2020). Inflammation brought on by oxidative stress intensifies oxidative damage and, if the balance is not restored, may result in apoptosis. Understanding this relationship is necessary for therapeutic efforts to reduce oxidative stress and chronic inflammation.

**FIGURE 10:**
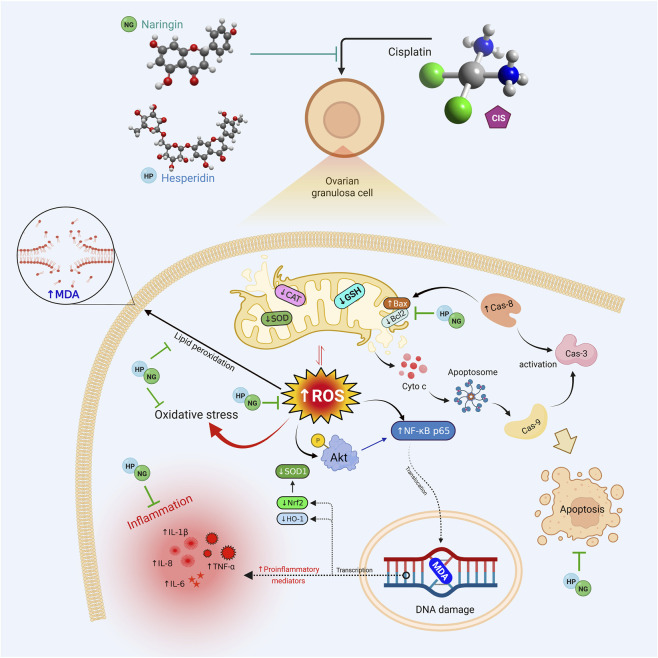
Proposed pathways through which NG or HP mitigate CIS-induced inflammation, oxidative stress, and apoptosis in ovarian granulosa cells.

As anticipated, consistent clustering patterns in all data sets in PCA and correlation heatmap confirmed the above-mentioned outcomes, showing a clear separation between the CIS-treated group and the treatment groups, whereas the NG- and HP-treated groups clustered closer to the normal controls. The clustering pattern indicates that NG and HP effectively restored the global molecular profile disrupted by CIS, suggesting coordinated regulation of multiple interconnected signaling pathways. Multivariate analysis specifically validates the involvement of oxidative stress-responsive (Nrf2/HO-1), inflammatory (NF-κB/TNF-α), and apoptosis-related (Bax/Bcl-2/Cas-3) signaling pathways, which together modulate ovarian granulosa cell survival and function. Similarly, volcano plot analysis showed that variables related to oxidative damage, inflammation, and apoptosis were the most significantly altered biological processes in CIS-exposed ovarian tissues compared to the other groups. The differential expression of oxidative stress biomarkers (ROS, MDA, antioxidant enzymes), pro-inflammatory mediators (TNF-α, IL-6, NF-κB), and apoptotic markers (Bax, Bcl-2, Cas-3) highlights the prominent role of CIS-induced ovarian injury by excessive generation of reactive oxygen species, which activates NF-κB-dependent inflammatory cascades, and subsequently triggers the mitochondrial apoptotic pathway. On the contrary, NG or HP treatment shifted these variables toward the normal state, suggesting inhibition of oxidative and inflammatory signaling and the maintenance of mitochondrial integrity and cellular viability. Furthermore, the tight clustering of antioxidant markers with anti-apoptotic mediators and the inverse correlation between oxidative stress indices and inflammatory/apoptotic biomarkers in the correlation heatmap add weight to the evidence for a tightly interconnected regulatory network. These results suggest that inhibition of oxidative stress induced by NG and HP may be the upstream event limiting inflammatory activation and downstream apoptotic signaling, thereby preserving the architecture of ovarian tissue and the function of granulosa cells. A proposed model for mechanisms involved in NG or HP protection against CIS-induced inflammation, oxidative stress, and apoptosis in ovarian granulosa cells is presented in Figure 10.

Interestingly, the pathways targeted in the present study are tightly interconnected. ROS are important upstream regulators that connect inflammatory signaling to programmed cell death. Moderate ROS levels are a second messenger for IκB kinase (IKK) activation, which phosphorylates IκBα, leading to its degradation and subsequent nuclear translocation of NF-κB (Kumar Rajendran et al., 2019). Activated NF-κB promotes the transcription of pro-inflammatory cytokines (TNF-α, IL-6), anti-apoptotic proteins (Bcl-2, Bcl-xl, c-IAPs), and redox-regulatory enzymes, thereby contributing to cell survival and amplification of inflammation (Pandey et al., 2025). Excess ROS shift NF-κB signaling from pro-survival to pro-death outcomes through oxidative damage to DNA, mitochondrial dysfunction, and sustained JNK/p53 activation. This results in permeabilization of the outer mitochondrial membrane, release of Cyt c, activation of Cas-9 and Cas-3, and, finally, apoptosis. ROS-NF-κB signaling is context-dependent, leading to survival at moderate stress levels and apoptosis when oxidative stress exceeds the cellular buffering capacity (Akcakavak et al., 2024). This threshold for redox-activity has important implications for cytotoxic selectivity.

Despite these promising results, several limitations of the present study warrant consideration. First, NG or HP alone at 80 μM did not affect basal cell viability (Figures 4B,C). But we did not comprehensively profile all oxidative, inflammatory, and apoptotic markers under flavonoid-only conditions. Since our experimental design was specifically designed to allow direct comparison between the injured (CIS) and rescued (CIS + flavonoid) states. Therefore, future studies should include treatment groups consisting solely of flavonoids to address this gap. Second, our protective assessments were limited to an *in vitro* human ovarian granulosa cell line, and this model-based limitation is compounded by the absence of histological and immunohistochemical evaluations, which precluded direct verification of tissue-level structural preservation and cellular integrity. Therefore, robust *in vivo* studies are necessary to demonstrate the protective efficacy of NG and HP against CIS-induced ovarian toxicity and their potential for clinical translation.

## 5 Conclusion

Our current findings substantiate that naringin and hesperidin are efficacious in mitigating CIS-induced granulosa cell damage by addressing interrelated pathways of oxidative stress, inflammation, mitochondrial dysfunction, and apoptosis. Their synchronized regulation of these molecular networks sustains cellular homeostasis and enhances granulosa cell viability under chemotherapeutic duress. These results underscore the potential of both flavonoids as viable adjuvants to mitigate ovarian damage associated with CIS treatment and to potentially save female fertility. Additional validation in sophisticated preclinical multi-cell line, *in vivo* studies, mechanistic target confirmation, and omics-based pathway analysis are recommended. In addition, clinical trials are necessary to determine their translational significance in fertility-preserving approaches for patients undergoing platinum-based chemotherapy.

## Data Availability

The original contributions presented in the study are included in the article/Supplementary Material, further inquiries can be directed to the corresponding authors.
