# Supplementary material for: Citrus flavonoids mitigate the cisplatin-induced ovarian toxicity via dual modulation of Nrf2/HO-1 pathway and NF-κB axis
Source: Front Pharmacol. 2026 Jul 17;17:1903801. doi: 10.3389/fphar.2026.1903801 (PMC13424696; doi:10.3389/fphar.2026.1903801)
Supplement: Supplementary file 1 [file Table1.docx]

**Table S1. Primers used in the study quantitative PCR assay.**

| **Gene** | **Sense (5′-3′)** | **Antisense (5′-3′)** |
| --- | --- | --- |
| *Akt1* | GAAGGACGGGAGCAGGCGGC | CCTCCTCCAGGCAGCCCCTT |
| *Akt2* | CCGGTGACAGACTGTGCCCTGT | CCCAATGAAGGAGCCGTCGCT |
| *Bax* | CGACGACTTCTCCCGCCGCTACCG C | CCGCATGCTGGGGCCGTACAG TTC C |
| *Bcl2* | TCCACCAAGAAGCTGAGCGAG | GTCCAGCCCATGATGGTT CT |
| *CAT* | CTTCGACCCAAGCAACATGC | GCGGTGAGTGTCAGGATAGG |
| *Ho-1* | AGGGAATTCTCTTGGCTGGC | GACAGCTGCCACATTAGGGT |
| *Nrf2* | CATCTACAAACGGGAATGTCTG | AGTGGATCTGCCAACTACTC |
| *SOD1* | GATGACTTGGGCAAAGGTGG | TACACCACAAGCCAAACGACT |
| *IL-1β* | GGATATGGAGCAACAACAAGTGG3 | ATGTACCAGTTGGGGGAACTG |
| *IL-6* | TCAATGAGGAGACTTGCCTG | GATGAGTTGTCATGTCCTGC3 |
| *IL-8* | TTTTGCCAAGGAGTGCTAAAGA | AACCCTCTGCACCCAGTTTTC |
| *TNF-α* | ACAAGCCTGTAGCCCATGTT | AAAGTAGACCTGCCCAGACT |
| *GAPDH* | GACAGTCAGCCGCATCTTCT | GCGCCCAATACGACCAAATC |

*Akt1, protein kinase B alpha (AKT1); Akt2, protein kinase B beta (AKT2); Bax, Bcl-2-associated X protein (BAX); Bcl2, B-cell lymphoma 2 (BCL2); CAT, catalase (CAT); HO-1, heme oxygenase-1 (HO-1); Nrf2, nuclear factor erythroid 2-related factor 2 (NRF2); SOD1, superoxide dismutase 1 (SOD1); IL-1β, interleukin-1 beta (IL-1β); IL-6, interleukin-6 (IL-6); IL-8, interleukin-8 (IL-8); TNF-α, tumor necrosis factor-alpha (TNF-α); and GAPDH, glyceraldehyde-3-phosphate dehydrogenase (GAPDH).*
